# A Review on the Nonlinear Dynamical System Analysis of Electrocardiogram Signal

**DOI:** 10.1155/2018/6920420

**Published:** 2018-05-02

**Authors:** Suraj K. Nayak, Arindam Bit, Anilesh Dey, Biswajit Mohapatra, Kunal Pal

**Affiliations:** ^1^Department of Biotechnology and Medical Engineering, National Institute of Technology, Rourkela, Odisha 769008, India; ^2^Department of Biomedical Engineering, National Institute of Technology, Raipur, Chhattisgarh 492010, India; ^3^Department of Electronics and Communication Engineering, Kaziranga University, Jorhat, Assam 785006, India; ^4^Vesaj Patel Hospital, Rourkela, Odisha 769004, India

## Abstract

Electrocardiogram (ECG) signal analysis has received special attention of the researchers in the recent past because of its ability to divulge crucial information about the electrophysiology of the heart and the autonomic nervous system activity in a noninvasive manner. Analysis of the ECG signals has been explored using both linear and nonlinear methods. However, the nonlinear methods of ECG signal analysis are gaining popularity because of their robustness in feature extraction and classification. The current study presents a review of the nonlinear signal analysis methods, namely, reconstructed phase space analysis, Lyapunov exponents, correlation dimension, detrended fluctuation analysis (DFA), recurrence plot, Poincaré plot, approximate entropy, and sample entropy along with their recent applications in the ECG signal analysis.

## 1. Introduction

**Figure 1:**
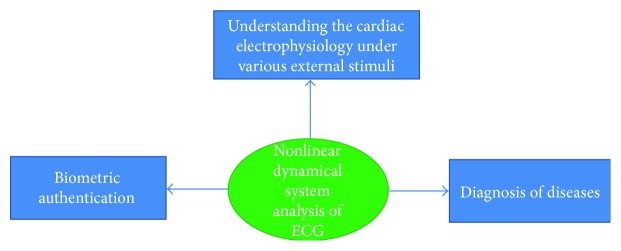
Various types of application of nonlinear dynamical system analysis of ECG.

In the last few decades, the ECG signals have been widely analyzed for the diagnosis of the numerous cardiovascular diseases [1, 2]. Apart from this, the ECG signals are processed to extract the RR intervals, which have been reported to divulge information about the influence of the autonomic nervous system activity on the heart through heart rate variability (HRV) analysis [3, 4]. HRV refers to the study of the variation in the time interval between consecutive heart beats and the instantaneous heart rate [5]. An important step in the analysis of the ECG signals is the extraction of the clinically relevant features containing all the relevant information of the original ECG signal and, hence, can act as the representative of the signal for further analysis [6, 7]. Features can be extracted from the ECG signals using the time-domain, frequency-domain, and joint time-frequency domain analysis methods including the nonlinear methods [7–9]. The analysis of the ECG signals using the nonlinear signal analysis methods has received special attention of the researchers in recent years [7–9]. The nonlinear methods of the ECG signal analysis derive their motivation from the concept of nonlinear dynamics [10, 11]. This may be attributed to the fact that the biomedical signals like ECG can be generated by the nonlinear dynamical systems [12]. A dynamical system is a system that changes over time [9]. However, a dynamical system may also be defined as an iterative physical system, which undergoes evolution over time in such a way that the future states of the system can be predicted using the preceding states [13]. Dynamical systems form the basis of the nonlinear methods of the signal analysis [14]. The highly explored nonlinear signal analysis methods include reconstructed phase space analysis, Lyapunov exponents, correlation dimension, detrended fluctuation analysis (DFA), recurrence plot, Poincaré plot, approximate entropy, and sample entropy. This study attempts to provide a theoretical background of the above-mentioned nonlinear methods and their recent applications (last 5 years) in the analysis of the ECG signal for the diagnosis of diseases, understanding the effect of external stimuli (e.g., low-frequency noise and music), and human biometric authentication (Figure 1).

## 2. Dynamical System

Dynamical systems form the basis of the nonlinear methods of signal analysis [15–17]. The study of the dynamical systems has found applications in a number of fields like physics [15–17], engineering [15], biology, and medicine [16]. A dynamical system can be defined as a system, whose state can be described by a set of time-varying (continuous or discrete) variables governed by the mathematical laws [17]. Such a system is said to be deterministic if the current values of time and the state variables can exactly describe the state of the system at the next instant of time. On the other hand, the dynamical system is regarded as stochastic, if the current values of time and the state variables describe only the probability of variation in the values of the state variables over time [18–20]. Dynamical systems can also be categorized either as linear or nonlinear systems. A system is regarded as linear when the change in one of its variable is proportional to the alteration in a related variable. Otherwise, it is regarded as nonlinear [18]. The main difference between the linear and the nonlinear systems is that the linear systems are easier to analyze. This can be attributed to the fact that the linear systems, unlike the nonlinear systems, facilitate the breaking down of the system into parts, performing analysis of the individual parts, and finally recombining the parts to obtain the solution of the system [21]. A set of coupled first-order autonomous differential equations ((1)) is used to mathematically describe the evolution of a continuous time dynamical system [22].

(1) $$\begin{array}{c} \frac{d\bar{x}\left(t\right)}{dt}=\bar{F}\left(\bar{x}\left(t\right),\bar{\mu }\right), \end{array}$$

where $\bar{x}\left(t\right)$ = vector representing the dynamical variables of the system, $\bar{\mu }$ = vector corresponding to the parameters, and $\bar{F}$ = vector field whose components are the dynamical rules governing the nature of the dynamical variables.

A system involving any nonautonomous differential equation in R*^n^* can be transformed into an autonomous differential equation in R^*n+* 1^ [23]. The forced Duffing-Van der Pol oscillator has been regarded as a well-known example of a nonlinear dynamical system, which is described by a second-order nonautonomous differential equation [14, 23].

(2) $$\begin{array}{c} \frac{d^{2}y}{{dt}^{2}}-\mu \left(1-y^{2}\right)\frac{dy}{dt}+y^{3}=f\cos wt, \end{array}$$

where *μ*, *f*, and *w* represent the parameters.

This nonautonomous differential equation can be converted into a set of coupled first-order autonomous differential equations (3), (4), and (5) by delineating 3 dynamical variables, that is, *x*_1_ = *y*, *x*_2_ = *dy*/*dt*, and *x*_3_ = *wt* [23].

(3) $$\begin{array}{c} \frac{{dx}_{1}}{dt}=x_{2}, \end{array}$$

(4) $$\begin{array}{c} \frac{{dx}_{2}}{dt}=\mu \left(1-{x_{1}}^{2}\right)x_{2}-{x_{1}}^{3}+f\cos x_{3}, \end{array}$$

(5) $$\begin{array}{c} \frac{{dx}_{3}}{dt}=w. \end{array}$$

The discrete time dynamical systems are described by a set of coupled first-order autonomous difference equations [14, 23, 24].

(6) $$\begin{array}{c} \bar{x}\left(n+1\right)\left(n+1\right)=\bar{G}\left(\bar{x}\left(n\right),\bar{\mu }\right), \end{array}$$

where $\bar{G}$ = vector describing the dynamical rules and *n* = integer representing time.

It is possible to obtain a discrete dynamical system from a continuous dynamical system through the sampling of its solution at a regular time interval *T*, in which the dynamical rule representing the relationship between the consecutive sampled values of the dynamical variables is regarded as a *time T map*. The sampling of the solution of a continuous dynamical system in the R*^n^* dimensional space at the consecutive transverse intersections with a R^*n−*1^ dimensional surface of the section also results in the formation of a discrete dynamical system. In this case, the dynamical rule representing the relationship between the consecutive sampled values of the dynamical variables is regarded as a *Poincaré map* or a *first return map*. For the forced Duffing-Van der Pol oscillator, the *Poincaré map* is equivalent to the *time T map* with *T* = 2*π*/*w* when a surface of section is defined by *x*_3_ = *θ*_0_ with *θ*_0_ ∈ (0, 2*π*) [14, 22, 23].

Generally, randomness is considered to be associated with noise (unwanted external disturbances like power line interference). However, it has been well reported in the last few decades that most of the dynamical systems are deterministic nonlinear in nature and their solutions can be statistically random as that of the outcomes of tossing an unbiased coin (i.e., head or tail) [23]. This statistical randomness is regarded as deterministic chaos, and it allows the development of models for characterizing the systems producing the random signals.

As per the reported literature, the random signals produced by noise fundamentally differ from the random signals produced from the deterministic dynamical systems with a small number of dynamical variables [25]. The differences between them cannot be analyzed using the statistical methods. Phase space reconstruction-based dynamical system analysis has been recommended by the researchers for this purpose [12].

## 3. Nonlinear Dynamical System Analysis Techniques

### 3.1. Reconstructed Phase Space Analysis of a Dynamical System

The phase space is an abstract multidimensional space, which is used to graphically represent all the possible states of a dynamical system [23]. The dimension of the phase space is the number of variables required to completely describe the state of the system [19, 26]. Its axes depict the values of the dynamical variables of the system [26]. If the actual number of variables governing the behaviour of the dynamical system is unknown, then the phase space plots are reconstructed by time-delayed embedding, which is based on the concept of Taken's theorem [19]. The theorem states that if the dynamics of a system is governed by a number of interdependent variables (i.e., its dynamics is multidimensional), and only one variable of the system, say, *x*, is accessible (i.e., only one dimension can be measured), then it is possible to reconstruct the complete dynamics of the system from the single observed variable *x* by plotting its values against itself for a certain number of times at a predefined time delay [27]. Fang et al. [28] have reported that the reconstructed phase spaces can be regarded as topologically equivalent to the original system and, hence, can recover the nonlinear dynamics of the system.

Let us consider that all the values of the observed variable *x* is represented by the vector $\bar{x}$.

(7) $$\begin{array}{c} \bar{x}=\left(x_{1},x_{2},x_{3},\ldots ,x_{n}\right), \end{array}$$

where *n* = number of points in the time series.

If *d* is the true/estimated embedding dimension of the system (i.e., number of variables governing the dynamics of the system), then each state of the system can be represented in the phase space by the *d*-dimensional vectors of the form ${\bar{v}}_{i}$ given as follows:

(8) $$\begin{array}{c} {\bar{v}}_{i}=\left(x_{1},x_{1+\tau },x_{1+2\tau },\ldots ,x_{1+\left(d-1\right)\tau }\right), \end{array}$$

where *τ* = time lag, and 1 ≤ *i* ≤ *n* − (*d* − 1)*τ*.

A total of *n* − (*d* − 1)*τ* number of such vectors are obtained, which can be arranged in a matrix **V** (9) [26, 27]. In matrix **V**, the row indices signify time, and the column indices refer to a dimension of the phase space.

This set of vectors forms the entire reconstructed phase space [12, 26].

(9) $$\begin{array}{c} V=\left[\begin{array}{c} {\bar{v}}_{1}\\ {\bar{v}}_{2}\\ \vdots \\ {\bar{v}}_{n-\left(d-1\right)\tau } \end{array}\right]=\left[\begin{array}{cccc} x_{1} & x_{1+\tau } & \cdots & x_{1+\left(d-1\right)\tau }\\ x_{2} & x_{2+\tau } & \cdots & x_{2+\left(d-1\right)\tau }\\ \vdots & \vdots & & \vdots \\ x_{n-\left(d-1\right)\tau } & x_{n-\left(d-2\right)\tau } & \cdots & x_{n} \end{array}\right], \end{array}$$

where the rows correspond to the *d*-dimensional phase space vectors and the columns represent the time-delayed versions of the initial *n* − (*d* − 1)*τ* points of the vector $\bar{x}$.

The two factors, namely, embedding dimension (*d*) and time delay (*τ*) play an important role during the reconstruction of the phase space of a dynamical system [29, 30]. The embedding dimension is determined using either the method of false nearest neighbours [12] or Cao's method [29] or empirically [30]. The false nearest neighbour method has been regarded as the most popular method for the determination of the optimal embedding dimension [31]. This method is based on the principle that the pair of points which are located very near to each other at the optimal embedding dimension *m* will remain close to each other as the dimension *m* increases further. Nevertheless, if *m* is small, then the points located far apart may appear to be neighbours due to projecting into a lower dimensional space. In this method, the neighbours are checked at increasing embedding dimensions until a negligible number of false neighbours are found while moving from dimension *m* to *m* + 1. This resulting dimension *m* is considered as the optimal embedding dimension.

The time delay is usually determined using either the first minimum of the average mutual information function (AMIF) [32] or first zero crossing of the autocorrelation function (ACF) [33] or empirically. The implementation of ACF is computationally convenient and does not require a large data set. However, it has been reported that the use of ACF is not appropriate for nonlinear systems, and hence AMIF should be used for the computation of the optimal time delay [34, 35]. For the discrete time signals, the AMIF can be defined as follows [36]:

(10) $$\begin{array}{c} AMI\left(X,Y\right)=\overset{M}{\underset{i=1}{\sum }}\overset{N}{\underset{j=1}{\sum }}P_{XY}\left(x_{i},y_{j}\right)\log\frac{P_{XY}\left(x_{i},y_{j}\right)}{P_{X}\left(x_{i}\right)P_{Y}\left(y_{j}\right)}, \end{array}$$

where *X* = {*x*_i_} and *Y* = {*y_j_*} are discrete time variables, *P*_*X*_(*x*_*i*_) is the probability of occurrence of *X*, *P*_*Y*_(*y*_*j*_) is the probability of occurrence of *Y*, and *P*_*XY*_(*x*_*i*_, *y*_*j*_) is the probability of occurrence of both *X* and *Y*.

**Figure 2:**
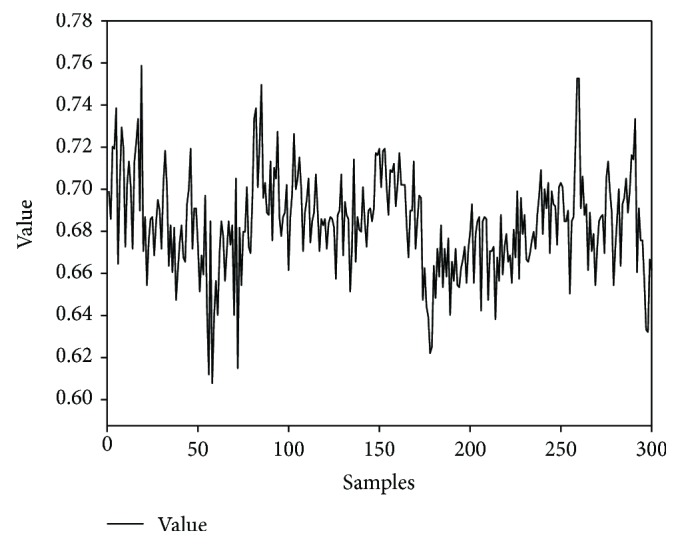
A representative RRI time series obtained from a 5 min ECG signal.

**Figure 3:**
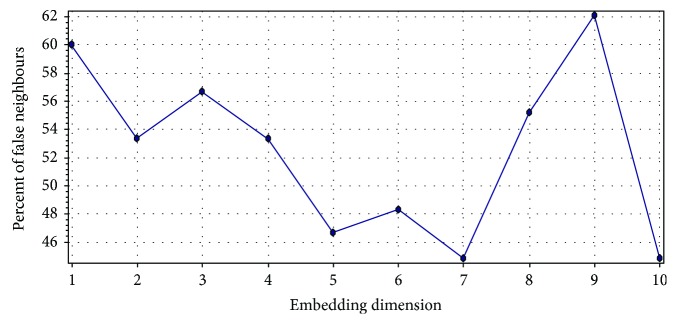
Computation of the optimal embedding dimension by the method of false nearest neighbours. The optimal embedding dimension was 7, and the corresponding percent false neighbour was 44.83%. The method of false nearest neighbour was implemented using Visual Recurrence Analysis freeware (V4.9, USA), developed by Kononov [37].

**Figure 4:**
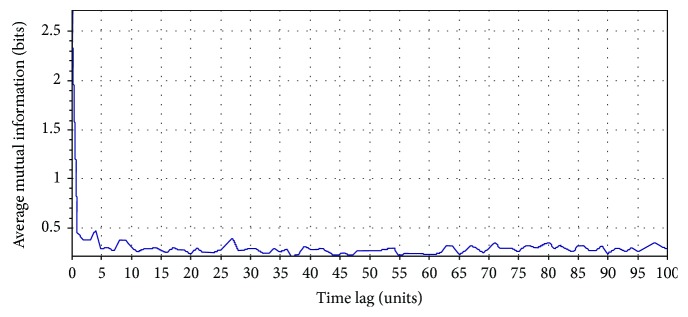
Optimal time delay computation by the first minimum of the AMIF. The first minimum of the AMIF was 2. The AMIF was calculated using Visual Recurrence Analysis freeware (V4.9, USA), developed by Kononov [37].

Let us consider an RR interval (RRI) time series extracted from the 5 min ECG recording of a person (Indian male volunteer of 27 years old) consuming cannabis (Figure 2). The ECG signal was acquired using the commercially available single lead ECG sensor (Vernier Software & Technology, USA) and stored into a laptop using a data acquisition device (NI USB 6009, National Instruments, USA). The sampling rate of the device was set at 1000 Hz. The RRI time series was extracted from the acquired ECG signal using Biomedical Workbench toolkit of LabVIEW (National Instruments, USA). The determination of the optimal value of the embedding dimension for this RRI time series by the method of false nearest neighbours is shown in Figure 3. The determination of the proper value of the time delay (by the first minimum of the AMIF) for the above-mentioned RRI time series has been shown in Figure 4.

**Figure 5:**
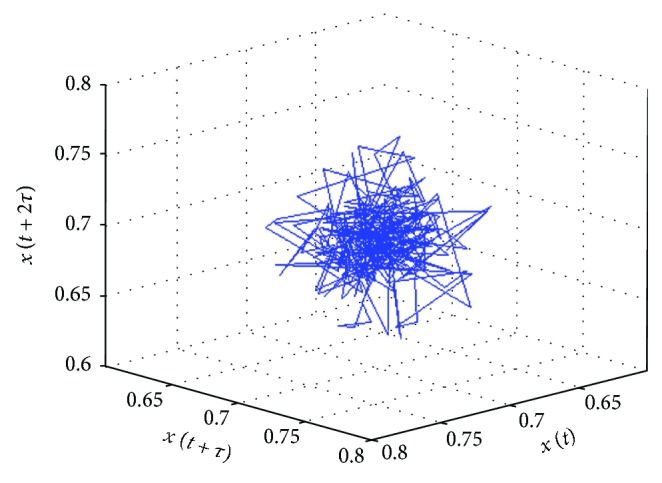
3D phase space attractor of an RRI time series. The attractor was plotted using the MATLAB Toolbox developed by Yang [39].

Each point in the reconstructed phase space of a system describes a potential state of the system. The system starts evolving from any point in the phase space (regarded as the initial state/condition of the system), following the dynamic trajectory determined by the equations of the system [19]. A dynamic trajectory describes the rate of change of the system's state with time. All the possible trajectories, for a given initial condition, form the flow of the system. Each trajectory occupies a subregion of the phase space, called as an attractor. An attractor can also be defined as a set of points (indicating the steady states) in the phase space, through which the system migrates over time [38]. The 3D attractor of the RRI time series (represented in Figure 2) has been shown in Figure 5.

Each attractor is associated with a basin of attraction, which represents all the initial states/conditions of the system that can go to that particular attractor [38]. Attractors can be points, curves, manifolds, or complicated objects, known as strange attractors. A strange attractor is an attractor having a noninteger dimension.

### 3.2. Lyapunov Exponents

The nonlinear dynamical systems are highly sensitive to the initial conditions, that is, a small change in the state variables at an instant will cause a large change in the behaviour of the system at a future instant of time. This is visualized in the reconstructed phase space as the adjacent trajectories that diverge widely from the initial close positions or converge. Lyapunov exponents are a quantitative measure of the average rate of this divergence or convergence [40]. They provide an estimation of the duration for which the behaviour of a system is predictable before chaotic behaviour prevails [9]. Positive Lyapunov exponent values indicate that the phase space trajectories are diverging (i.e., the closely located points in the initial state are rapidly separating from each other in the *i*th direction) and the system is losing its predictability, exhibiting chaotic behaviour [41, 42]. On the other hand, the negative Lyapunov exponent values are representatives of the average rate of the convergence of the phase space trajectories. For example, in a three-dimensional system, the three Lyapunov exponents provide information about the evolution of the volume of a cube and their sum specifies how a hypercube evolves in a multidimensional attractor. The sum of the positive Lyapunov exponents represents the rate of spreading of the hypercube, which in turn, indicates the increase in unpredictability per unit time. The largest positive (dominant) Lyapunov exponent mainly governs its dynamics [43].

If ‖*δx*_*i*_(0)‖ and ‖*δx*_*i*_(*t*)‖ represent the Euclidean distance between two neighbouring points of the phase space in the *i*th direction at the time instances of 0 and *t*, respectively, then, the Lyapunov exponent can be defined as the average growth *λ*_*i*_ of the initial distance ‖*δx*_*i*_(0)‖ [23, 44].

(11) $$\begin{array}{c} \frac{\left\|\delta x_{i}\left(t\right)\right\|}{\left\|\delta x_{i}\left(0\right)\right\|}=e^{\lambda_{i}t}\left(t\to \infty \right),\\ \text{or }\lambda_{i}=\lim_{t\to \infty }\frac{1}{t}\log\frac{\left\|\delta x_{i}\left(t\right)\right\|}{\left\|\delta x_{i}\left(0\right)\right\|}, \end{array}$$

where *λ*_*i*_ is the average growth of the initial distance ‖*δx*_*i*_(0)‖.

The dimensionality of the dynamical system decides the number of Lyapunov exponents, that is, if the system is defined in R*^m^*, then it possesses *m* Lyapunov exponents (*λ*_1_ ≥ *λ*_2_ ≥ ,…, *λ*_*m*_). The complete set of Lyapunov exponents can be described by considering an extremely small sphere of initial conditions having *m* dimensions, which is fastened to a reference phase space trajectory. If *P_i_*(*t*) represents the length of the *i*th axis, and the axes are arranged in the order of the fastest to the slowest growing axes, then 12 denotes the complete set of Lyapunov exponents arranged in the order of the largest to the smallest exponent [23].

(12) $$\begin{array}{c} \lambda_{i}=\lim_{t\to \infty }\frac{1}{t}\log\left(\frac{P_{i}\left(t\right)}{P_{i}\left(0\right)}\right), \end{array}$$

where *i* = 1, 2,…, *m*.

The divergence of the vector field of a dynamical system is identical to the sum of all its Lyapunov exponents (13). Hence, the sum of all the Lyapunov exponents is negative in case of the dissipative systems. Also, one of the Lyapunov exponents is zero for the bounded trajectories, which do not approach a fixed point.

(13) $$\begin{array}{c} \overset{m}{\underset{i=1}{\sum }}\lambda =\bar{\nabla }\cdot \bar{F}, \end{array}$$

where $\bar{F}$ represents the vector field of a dynamical system.

Lyapunov exponents can be calculated from either the mathematical equations describing the dynamical systems (if known) or the observed time series [45]. Usually, two different types of methods are used for obtaining the Lyapunov exponents from the observed signals. The first method is based on the concept of the time-evolution of nearby points in the phase space [46]. However, this method enables the evaluation of the largest Lyapunov exponent only. The other method is dependent on the computation of the local Jacobi matrices and estimates all the Lyapunov exponents [47]. All the Lyapunov exponents (in vector form) of a particular system constitute the Lyapunov spectra [45].

### 3.3. Correlation Dimensions

The geometrical objects possess a definite dimension. For example, a point, a line, and a surface have dimensions of 0, 1, and 2, respectively [9]. This notion has led to the development of the concept of fractal dimension. A fractal dimension refers to any noninteger dimension possessed by the set of points (representing a dynamical system) in a Euclidean space. The determination of the fractal dimension plays a significant role in the nonlinear dynamic analysis. This may be attributed to the fact that the strange attractors are fractal in nature and their fractal dimension indicates the minimum number of dynamical variables required to describe the dynamics of the strange attractors. It also quantitatively portrays the complexity of a nonlinear system. The higher is the dimension of the system; the more is the complexity. The commonly employed method for the determination of the dimension of a set is the measurement of the *Kolmogorov capacity* (i.e., box-counting dimension). This method covers the set with tiny cells/boxes (squares for sets embedded in 2D and cubes for sets embedded in 3D space) having size *ϵ*. The dimension *D* can be defined as follows [23]:

(14) $$\begin{array}{c} D=\lim_{\varepsilon \to 0}\frac{\log\left(M\left(\varepsilon \right)\right)}{\log\left(1/\varepsilon \right)}, \end{array}$$

where *M*(*ϵ*) is the number of the tiny boxes containing a part of the set.

**Figure 6:**
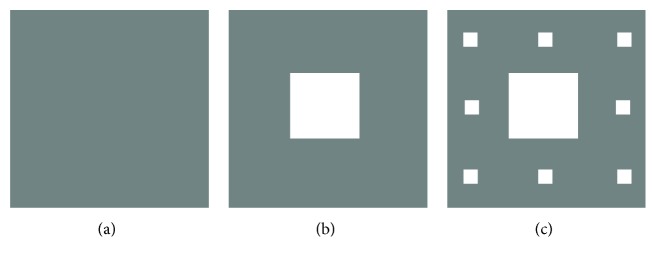
Illustration of the first 3 stages during the construction of a Cantor set in 2D: (a) *n* = 0, (b) *n* = 1, and (c) *n* = 2 [48].

The mathematical example of a set possessing noninteger fractal dimension is a Cantor set. A Cantor set can be defined as the limiting set in a sequence of sets [48]. Let us consider a Cantor set in 2D, characterized by the below mentioned sequence of sets. At stage *n* = 0 (Figure 6(a)), let S_0_ designates a square having sides of length *l*. The square S_0_ is divided into 9 uniform squares of size *l*/3, and the middle square is removed at stage *n* = 1 (Figure 6(b)). This set of squares is regarded as S_1_. At stage *n* = 2, each square of set S_1_ is further divided into 9 squares of size *l*/9 and the middle squares are removed, which constitute the set S_2_ (Figure 6(c)). When this process of subdivision and removal of squares is continued to get the sequence of sets S_0_, S_1_, and S_2_, then the Canter set is the limiting set defined by $S=\lim_{n\to \infty }\;S_{n}$. The *Kolmogorov capacity*-based dimension of this Cantor set can be calculated easily using the principle of mathematical induction as described below. When *n* = 0, S_0_ consists of a square of size *l,* and hence, *ϵ* = *l* and *M*(*ϵ*) = 1. When *n* = 1, S_1_ comprises of 8 squares of size *l*/3. Therefore, *ϵ* = *l*/3 and *M*(*ϵ*) = 8. At *n* = 2, S_2_ is made of 64 squares of size *l*/9. Therefore, *ϵ* = (*l*/3)^2^ and *M*(*ϵ*) = 8^2^. Thus, the fractal dimension of the Cantor set is given as follows:

(15) $$\begin{array}{c} D=\lim_{\varepsilon \to 0}\frac{\log\left(M\left(\varepsilon \right)\right)}{\log\left(1/\varepsilon \right)}=\lim_{n\to \infty }\frac{\log\left(8^{n}\right)}{\log\left(l/3^{n}\right)}=1.892, \end{array}$$

where the fractal dimension < 2 suggests that the Cantor set does not completely fill an area in the 2D space.

However, the *Kolmogorov capacity*-based dimension measurement does not describe whether a box contains many points or few points of the set. To describe the inhomogeneities or correlations in the set, Hentschel and Procaccia defined the dimension spectrum [49].

(16) $$\begin{array}{c} D_{q}=\lim_{r\to 0}\frac{1}{q-1}\frac{\log\sum_{i=1}^{M\left(r\right)}P_{i}^{q}}{\log r},\;q=0,1,2,\ldots , \end{array}$$

where *M*(*r*) = number of *m*-dimensional boxes of size *r* required to cover the set, *p_i_* = *N_i_*/*N* is the probability that the *i*th box contains a point of the set, *N* is the total number of points in the set, and *N_i_* is the number of points of the set contained by the *i*th box.

It can be readily inferred that the *Kolmogorov capacity* is equivalent to *D*_0_. The dimension *D*_1_ defined by taking the limit *q* → 1 in 16 is regarded as the information dimension.

(17) $$\begin{array}{c} D_{1}=\lim_{q\to 1}D_{2}=\lim_{r\to 0}\frac{\sum_{i=1}^{M\left(r\right)}P_{i}\log P_{i}}{\log r}, \end{array}$$

where the dimension *D*_2_ is the known as the correlation dimension.

The correlation dimension can be expressed as follows:

(18) $$\begin{array}{c} D_{2}=\lim_{r\to 0}\frac{\log C\left(r\right)}{\log r}, \end{array}$$

where *C*(*r*) = ∑_*i*=1_^*M*(*r*)^*p*_*i*_^2^ is the correlation sum. It represents the probability of occurrence of two points of the set in a single box.

The correlation dimension signifies the number of the independent variables required to describe the dynamical system [50]. A widely used algorithm for the computation of the correlation dimension (*D*_2_) from a finite, discrete time series was introduced by Grassberger and Procaccia [51]. It was based on the assumption that the probability of occurrence of two points of the set in a box of size *r* is approximately same as the probability that the two points of the set are located at a distance *ρ* ≤ *r*. Using this assumption, the correlation sum can be computed as given as follows:

(19) $$\begin{array}{c} C\left(r\right)\approx \frac{\sum_{i=1,j>i}^{N}\Theta \left(r-\rho \left(x_{i},y_{i}\right)\right)}{1/2N\left(N-1\right)}, \end{array}$$

where Θ is the Heaviside function and can be defined as

(20) $$\begin{array}{c} \Theta \left(u\right)=\left\{\begin{array}{ll} 0, & \text{if }u\leq 0,\\ 1, & \text{if }u\geq 0. \end{array}\right. \end{array}$$

**Figure 7:**
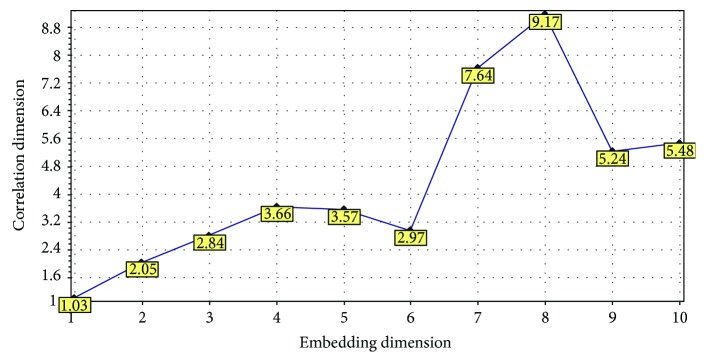
Correlation dimensions of the reconstructed phase space plot of RRI time series at different embedding dimensions. The correlation dimensions were calculated using Visual Recurrence Analysis freeware (V4.9, USA), developed by Kononov [37].

Practically, it is not possible to achieve the limit *r* → 0 that is used in the definition of the correlation dimension (18). Hence, Grassberger and Procaccia [51] proposed the approximate calculation of the correlation sum *C*(*r*) (19) for a number of values of *r* and then deducing the correlation dimension from the slope of the linear fitting in the linear region of the plot of log(*C*(*r*)) versus log(*r*). The correlation dimension of the reconstructed phase space plot of a dynamical system varies with its embedding dimension. The correlation dimensions of the reconstructed phase space plot of the aforementioned RRI time series at different embedding dimensions have been shown in Figure 7.

### 3.4. Detrended Fluctuation Analysis (DFA)

The detection of long-range correlation of a nonstationary time series data requires the distinction between the trends and long-range fluctuations innate to the data. Trends are resulted due to external effects, for example, the seasonal alteration in the environmental temperature values, which exhibits a smooth and monotonous or gradually oscillating behaviour. Strong trends in the time series can cause the false discovery of long-range correlations in the time series if only one nondetrending technique is used for its analysis or if the outcomes of a method are misinterpreted. In recent years, DFA is explored for identifying long-range correlations (autocorrelations) of the nonstationary time series data (or the corresponding dynamical systems) [52]. This may be attributed to the ability of DFA to systematically eliminate the trends of different orders embedded into the data [52]. It provides an insight into the natural fluctuation of the data as well as into the trends in the data. DFA estimates the inherent fractal-type correlation characteristics of the dynamical systems, where the fractal behaviour corresponds to the scale invariance (or self-similarity) among the various scales [9]. The method of DFA was first proposed by Peng et al. [53] for the identification and the quantification of long-range correlations in DNA sequences. It was developed for detrending the variability in a sequence of events, which in turn, can divulge information about the long-term variations in the dataset. Since its inception, DFA has found applications in the study of HRV [54], gait analysis [55, 56], stock market prediction [57, 58], meteorology [59], and geology [60–62]. DFA method has also been given alternative terminologies [61] by various researchers like “linear regression detrended scaled windowed variance” [63] and “residuals of regression” [64].

In order to implement DFA, the bounded time series *x_t_* (*t* ϵ *N*) is converted into an unbounded series *X_t_* [65].

(21) $$\begin{array}{c} X_{t}=\overset{t}{\underset{i=1}{\sum }}\left(x_{i}-\langle x_{i}\rangle \right), \end{array}$$

where *X_t_* = cumulative sum and 〈*x*_*i*_〉 = mean of the time series *x_t_* in the window *t.*

The unbounded time series *X_t_* is then split into a number of portions of equal length *n*, and a straight line fitting is performed to the data using the method of least square fitting. The fluctuation (i.e., the root-mean-square variation) for every portion from the trend is calculated using [9]

(22) $$\begin{array}{c} F\left(n\right)=\sqrt{\frac{1}{n}\overset{n}{\underset{i=1}{\sum }}{\left(X_{i}-a_{i}-b\right)}^{2}}, \end{array}$$

where *a_i_* and *b* indicate the slope and intercept of the straight line fitting, respectively, and *n* is the split-unbounded time series portion length.

**Figure 8:**
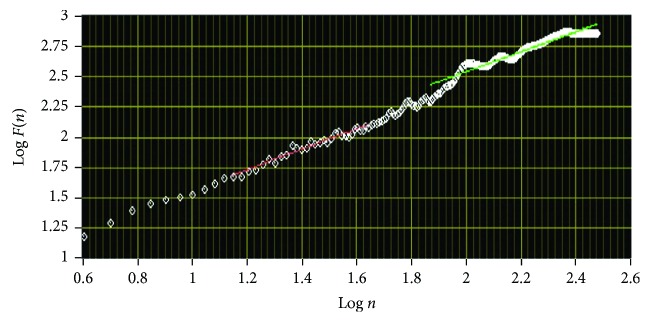
Log-log graph of *F*(*n*) versus *n* for RRI time series. The graph was plotted using Biomedical Workbench toolkit of LabVIEW (National Instruments, USA).

Finally, the log-log graph of *F*(*n*) versus *n* is drawn (Figure 8), where the statistical self-similarity of the signal is represented by the straight line on this graph, and the scaling exponent *α* is obtained from the slope of the line. The self-similarity is indicated as *F*(*n*) ∝ *n*^*α*^. The fluctuation exponent *α* has different values for different types of data (e.g., *α*~1/2 for the uncorrelated white noise and *α* > 1/2 for the correlated processes) [66, 67].

### 3.5. Recurrence Plot and Recurrence Quantification Analysis

The dynamical features (e.g., entropy, information dimension, dimension spectrum, and Lyapunov exponents) of a time series can be computed using various methods [68]. However, most of these methods assume that the time series data is obtained from an autonomous dynamical system. In other words, the evolution equation of the time series data does not involve the time explicitly. Further, the time series data should be longer than the characteristic time of the underlying dynamical system. In this regard, the recurrence plot reported by Eckmann et al. [68] has emerged as an important method for the analysis of the dynamical systems and provides useful information even when the aforementioned assumptions are not satisfied. If ${\left\{{\bar{x}}_{i}\right\}}_{i=1}^{N}$ represents the phase-space trajectory of a dynamical system in a *d*-dimensional space, then the recurrence plot can be defined as an array of points positioned at the places (*i*, *j*) in a **N** × **N** square matrix (23) such that ${\bar{x}}_{j}$ is approximately equal to ${\bar{x}}_{i}$ as described by 24 [68–70].

(23) $$\begin{array}{c} R_{i,j}\left(\varepsilon \right)=\left\{\begin{array}{ll} 1, & x_{i}\approx x_{j},\\ 0, & x_{i}\neq x_{j}, \end{array}\right. \end{array}$$

(24) $$\begin{array}{c} \left|{\bar{x}}_{j}-{\bar{x}}_{i}\right|\leq \varepsilon , \end{array}$$

where *ε* = acceptable distance (error) between ${\bar{x}}_{i}$ and ${\bar{x}}_{j}$. This *ε* is required because many systems often do not recur exactly to a previous state but just approximately.

**Figure 9:**
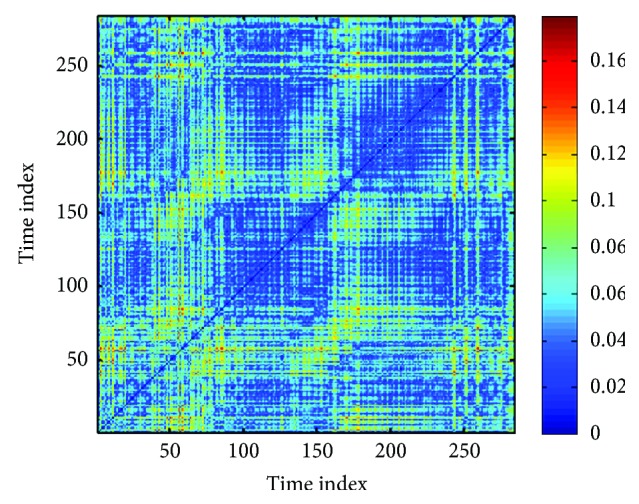
Recurrence plot of an RRI time series. The recurrence plot was generated using the MATLAB Toolbox developed by Yang [39].

Recurrence plot divulges natural time correlation information at times *i* and *j*. In other words, it evaluates the states of a system at times *i* and *j* and indicates the existence of similarity by placing a dot (corresponding to **R**_*i*,*j*_ = 1) in the recurrence plot. The recurrence plot of the RRI time series present in Figure 2 has been shown in Figure 9.

The main advantage of the recurrence plot is that it does not require any mathematical transformation or assumption [69]. But the drawback of this method lies in the fact that the information provided is qualitative. To overcome this limitation, several measures of complexity that quantify the small-scale structures in the recurrence plot have been proposed by many researchers, regarded as recurrence quantification analysis (RQA) [71]. These measures are derived from the recurrence point density as well as the diagonal and the vertical line structures of the recurrence plot. The calculation of these measures in small windows, passing along the line of identity (LOI) of the recurrence plot, provides information about the time-dependent behaviour of these variables. Several studies have reported that the RQA variables can detect the bifurcation points like the chaos-order transitions [72]. The vertical structures in the recurrence plot have been reported to represent the intermittency and the laminar states. The RQA variables, corresponding to the vertical structures, enable the detection of the chaos-chaos transition [71]. The following discussion introduces the RQA parameters along with their potentials in the identification of the changes in the recurrence plot.

(i) Recurrence rate (RR) or percent recurrences: RR is the simplest variable of the RQA. It is a measure of the density of the recurrence points in the recurrence plot. Mathematically, it can be defined as 25, which is related to the correlation sum (19) except LOI, which is not included.
  (25) $$\begin{array}{c} RR\left(\varepsilon \right)=\frac{1}{N^{2}}\overset{N}{\underset{i,j=1}{\sum }}R_{i,j}\left(\varepsilon \right), \end{array}$$
  where **R**_*i*,*j*_(*ε*) is the recurrence matrix and *N* is the length of the data series.
(ii) Average number of neighbours: It is defined by 26 and represents the average number of neighbours possessed by each point of the trajectory in its *ε*-neighbourhood.
  (26) $$\begin{array}{c} N_{n}\left(\varepsilon \right)=\frac{1}{N}\overset{N}{\underset{i,j=1}{\sum }}R_{i,j}\left(\varepsilon \right), \end{array}$$
  where *N*_n_ is the number of (nearest) neighbours.
(iii) Determinism: The recurrence plot comprises of diagonal lines. The uncorrelated, stochastic, or chaotic processes exhibit either no diagonal lines or very short diagonal lines. On the other hand, the deterministic processes are associated with longer diagonals and less number of isolated recurrence points. The ratio of the number of recurrence points forming diagonal structures (having length ≥ *l*_min_) to the total number of recurrence points is regarded as determinism (DET) or predictability of the system (27). The threshold *l*_min_ is used to exclude the diagonal lines which are produced by the tangential motion of the phase space trajectory.
  (27) $$\begin{array}{c} DET=\frac{\sum_{l=l_{\min}}^{N}lP\left(l\right)}{\sum_{l=1}^{N}lP\left(l\right)}, \end{array}$$
  where *P*(*l*) = ∑_*i*,*j*=1_^*N*^(1 − **R**_*i*−1,*j*−1_(*ε*))(1 − **R**_*i*+*l*,*j*+*l*_(*ε*))∏_*k*=0_^*l*−1^**R**_*i*+*k*,*j*+*k*_(*ε*) represents the histogram of diagonal lines of length *l*.
(iv) Divergence: Divergence (DIV) is the inverse of the longest diagonal line appearing in the recurrence plot (28). It corresponds to the exponential divergence of the phase space trajectory, that is, when the divergence is more, the diagonal lines are shorter, and the trajectory diverges faster.
  (28) $$\begin{array}{c} DIV=\frac{1}{L_{\max}}=\frac{1}{\max\left({\left\{l_{i}\right\}}_{i=1}^{N_{l}}\right)}, \end{array}$$
  where *L*_max_ is the length of the longest diagonal line.
(v) Entropy: Entropy (ENTR) is the Shannon entropy of the probability *p*(*l*) of finding a diagonal line of length *l* in the recurrence plot (29). It indicates the complexity of the recurrence plot in respect of the diagonal lines. For example, the uncorrelated noise possesses a small value of entropy, which suggests its low complexity.
  (29) $$\begin{array}{c} ENTR=-\overset{N}{\underset{l=l_{\min}}{\sum }}p\left(l\right)\ln\left(p\left(l\right)\right), \end{array}$$
  where *p*(*l*) is the probability of finding a diagonal line of length *l*.
(vi) RATIO: It is the ratio of the determinism and the recurrence rate (30). It has been reported to be useful for identifying the transitions in the dynamics of the system.
  (30) $$\begin{array}{c} RATIO=N^{2}\frac{\sum_{l=l_{\min}}^{N}lP\left(l\right)}{{\left(\sum_{l=1}^{N}lP\left(l\right)\right)}^{2}}, \end{array}$$
  where *P*(*l*) = number of diagonal lines of length *l*.
(vii) Laminarity: Laminarity (LAM) is the ratio of the number of recurrence points forming vertical lines to the total number of recurrence points in the recurrence plot (31). LAM has been reported to provide information about the occurrence of the laminar states in the system. However, it does not describe the length of the laminar states. The value of LAM decreases if more number of single recurrence points are present in the recurrence plot than the vertical structures.
  (31) $$\begin{array}{c} LAM=\frac{\sum_{v=v_{\min}}^{N}vP\left(v\right)}{\sum_{v=1}^{N}vP\left(v\right)}, \end{array}$$
  where *P*(*v*) = ∑_*i*,*j*=1_^*N*^(1 − **R**_*i*,*j*_)(1 − **R**_*i*,*j*+*v*_)∏_*k*=0_^*v*−1^**R**_*i*,*j*+*k*_ is number of vertical lines of length *v*.
(viii) Trapping time: Trapping time (TT) is an estimate of the average length of the vertical structures, defined by 32. It indicates the average time for which the system will abide by a specific state. The computation of TT requires the consideration of a minimum length *v*_min_.
  (32) $$\begin{array}{c} TT=\frac{\sum_{v=v_{\min}}^{N}vP\left(v\right)}{\sum_{v=v_{\min}}^{N}P\left(v\right)}, \end{array}$$
  where *v*_min_ is the predefined minimum length of a vertical length.
(ix) Maximum length of the vertical lines: The maximum length of the vertical lines (*V*_max_) in the recurrence plot can be defined as follows:
  (33) $$\begin{array}{c} V_{\max}=\max\left({\left\{v_{l}\right\}}_{l=1}^{N_{v}}\right), \end{array}$$
  where *N*_*v*_ is the absolute number of vertical lines.

### 3.6. Poincaré Plot

**Figure 10:**
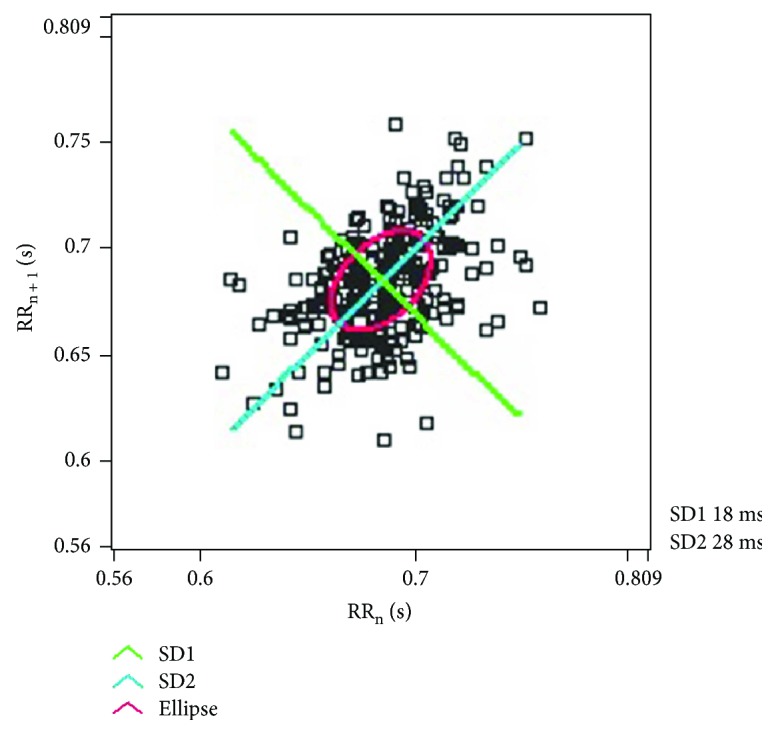
The Poincaré plot of the RRI time series represented in Figure 2. The plot was generated using Biomedical Workbench toolkit of LabVIEW (National Instruments, USA).

A Poincaré plot is a plot that enables the visualization of the evolution of a dynamical system in the phase space and is useful for the identification of the hidden patterns. It facilitates the reduction of dimensionality of the phase space and simultaneously converts the continuous time flow into a discrete time map [9]. The Poincaré plot varies from the recurrence plot in the sense that Poincaré plot is defined in a phase space, whereas, the recurrence plot is created in the time space. In the recurrence plot, the points represent the instances when the dynamical system traverses approximately the same section of the phase space [9]. On the other hand, the Poincaré plot is generated by plotting the current value of the RR interval (RR_*n*_) against the RR interval value preceding it (RR_*n*+1_) [73, 74]. Hence, the Poincaré plot takes into account only the length of the RR intervals but not the amount of the RR intervals that occur [75]. The Poincaré plot is also named as scatter plot or scattergram, return map, and Lorentz plot [76]. The Poincaré plot of the aforementioned RRI time series has been shown in Figure 10.

Two important descriptors of the Poincaré plot are SD1 and SD2. SD1 refers to the standard deviation of the projection of the Poincaré plot on the line normal to the line of identity (i.e., *y* = −*x*), whereas, the projection on the line of identity (i.e., *y* = *x*) is regarded as SD2 [77]. The ratio of SD1 and SD2 is named as SD12. The Poincaré plot has been reported to divulge information about the cardiac autonomic activity [78, 79]. This can be attributed to the fact that SD1 provide information on the parasympathetic activity, whereas, SD2 is inversely related to sympathetic activity [80].

Apart from the above-mentioned dynamical system analysis methods, entropy-based measures such as approximate entropy (ApEn) and sample entropy (SaEn) have also been studied for the analysis of nonstationary signals [9]. These measures have been proposed to reduce the number of points required to obtain the dimension or entropy of low-dimensional chaotic systems and to quantify the changes in the process entropy. However, the methodological drawbacks of ApEn have been pointed out by Richman and Moorman and Costa et al. [9, 81, 82]. SaEn has also suffered from criticism for not completely characterizing the complexity of the signal [9, 83].

## 4. Applications of Nonlinear Dynamical System Analysis Methods in ECG Signal Analysis

### 4.1. Applications of Phase Space Reconstruction in ECG Signal Analysis

The phase space reconstruction has found a wide range of applications in the field of research, such as wind speed forecasting for wind farms [84], analyzing molecular dynamics of polymers [85], river flow prediction in urban area [86], and biosignal (such as ECG and EEG) analysis [28]. Among the applications related to biosignal analysis, many extensive studies have been performed for the analysis of ECG signals [87].

The different types of cardiac arrhythmias include ventricular tachycardia, atrial fibrillation, and ventricular fibrillation. Al-Fahoum and Qasaimeh [12] have reported the development of a simple ECG signal processing algorithm which employs reconstructed phase space for the classification of the different types of arrhythmia. The regions occupied by the ECG signals (belonging to the different types of arrhythmias) in the reconstructed phase space were used to extract the features for the classification of the arrhythmias. The authors reported the occurrence of 3 regions in the reconstructed phase space, which were representative of the concerned arrhythmias. Hence, 3 simple features were computed for the purpose of arrhythmia classification. The performance of the proposed algorithm was verified by classifying the datasets from the MIT database. The algorithm was able to achieve a sensitivity of 85.7–100%, a specificity of 86.7–100%, and an overall efficiency of 95.55%. Sayed et al. [88] have proposed the use of a novel distance series transform domain, which can be derived from the reconstructed phase space of the ECG signals, for the classification of the five types of arrhythmias. The transform space represents the manner in which the successive points of the original reconstructed phase space travel nearer or farther from the origin of the phase space. A combination of the raw distance series values and the parameters of the autoregressive (AR) model, the amplitude of the discrete Fourier transform (DFT), and the coefficients of the wavelet transform was used as the features for classification using K-nearest neighbour (K-NN) classifier. The authors have reported that the proposed method outperformed the state-of-the-art methods of classification with an extraordinary accuracy of 98.7%. The sensitivity and the specificity of the classifier were 99.42% and 98.19%, respectively. Based on the results, the authors suggested that their proposed method can be used for the classification of the ECG signals. The recent studies performed in the last 5 years for arrhythmia detection using phase space analysis of the ECG signals have been tabulated in Table 1.

Sleep apnoea is a kind of sleep disorder, where a distinct short-term cessation of breathing for >10 sec is observed when the person is sleeping [92]. It can be categorized into 3 categories, namely, obstructive sleep apnoea, central sleep apnoea, and mixed sleep apnoea. Sleep apnoea results in symptoms like daytime sleeping, irritation, and poor concentration [93]. Jafari reported the extraction of the features from the reconstructed phase space of the ECG signals and the frequency components of the heart rate variability (HRV) (i.e., very low-frequency (VLF), low-frequency (LF), and high-frequency (HF) components) for the detection of the sleep apnoea [93]. The extracted features were subjected to SVM-based classification. For the sleep apnoea dataset provided by Physionet database, the proposed feature set exhibited a classification accuracy of 94.8%. Based on the results, the author concluded that the proposed method can help in improving the efficiency of sleep apnoea detection systems.

Syncope, also known as fainting, refers to the unanticipated and the temporary loss of consciousness [94]. This is due to the malfunctioning of the autonomic nervous system (ANS), which is responsible for the regulation of the heart rate and blood pressure [95]. Syncope is characterized by a reduction in blood pressure and bradycardia [95]. It is diagnosed using a medical procedure known as head-up tilt test (HUTT) that varies from 45 to 60 min [96]. Since the test has to be carried out for a long time, it is unsuitable for the physically weak patients as they cannot complete the test. Thus, methods have been proposed to reduce the duration of the test through the prediction of the HUTT results by analyzing cardiovascular signals (e.g., ECG and blood pressure) acquired during HUTT. Khodor et al. [96] proposed a novel phase space analysis algorithm for the detection of syncope. HUTT was carried out for 12 min, and the ECG signals were acquired simultaneously. RR intervals were extracted from the ECG signals, and the phase space plots were reconstructed. Features were extracted from the phase space plot (such as phase space density) and recurrence quantification analysis. Statistically significant parameters were determined using Mann–Whitney test, which were further used for the SVM-based classification. Sensitivity and specificity of 95% and 47% were achieved. In 2015, the same group further reported the acquisition of arterial blood pressure signal along with the ECG signal during the HUTT for the detection of syncope [95]. Features were derived from the phase space analysis of the acquired signals, and important predictors were identified using the relief method [97]. The K-NN-based classification was performed, and a sensitivity of 95% and a specificity of 87% were achieved. Based on the results, the authors suggested that a bivariate analysis may be performed instead of univariate analysis to predict the outcome of HUTT with improved performance.

In recent years, ECG is being widely explored as a biometric to secure body sensor networks, human identification, and verification [98]. As compared to the other biometrics, it provides the advantage that it has to be acquired from a living body. In many previous studies related to the ECG-based biometric, features extracted from the ECG signals were amplitudes, durations, and areas of P, Q, R, S, and T waves [99–101]. However, the extraction of these features becomes difficult when the ECG gets contaminated by noise [102]. Wavelet analysis of the ECG signals was also attempted for the extraction of the ECG features for the identification of persons [103]. But, it required shifting of one ECG waveform with respect to the other for obtaining the best fit [104]. Recently, Fang and Chan proposed the development of an ECG biometric using the phase space analysis of the ECG signals [102]. The phase space plots were reconstructed from the 5 sec ECG signals, and the trajectories were condensed, single *course-grained* structure. The distinction between the *course-grained* structures was performed using the normalized spatial correlation (nSC), the mutual nearest point match (MNPM), and the mutual nearest point distance (MNPD) methods. The proposed strategy was tested on 100 volunteers using both single-lead and 3-lead ECG signals. The use of single-lead ECG signals resulted in the person identification accuracies of 96%, 95%, and 96% for MNPD, nSC, and MNDP methods, respectively, whereas, the accuracies increased up to 99%, 98%, and 98% for 3-lead ECG signals. Earlier, the same group had proposed the ECG biometric-based identification of humans by measuring the similarity or dissimilarity among the phase space portraits of the ECG signals [105]. In the experiment involving 100 volunteers, the person identification accuracies of 93% and 99% were achieved for single-lead and 3-lead ECG, respectively.

### 4.2. Applications of Lyapunov Exponents in ECG Signal Analysis

The concept of Lyapunov exponents has been employed to describe the dynamical characteristics of many biological nonlinear systems including cardiovascular systems. The versatility of the dominant Lyapunov exponents (DLEs) of the ECG signals was effectively applied by Valenza et al. [43] to characterize the nonlinear complexity of HRV in stipulated time intervals. The aforementioned study evaluated the HRV signal during emotional visual elicitation by using approximate entropy (ApEn) and dominant Lyapunov exponents (DLEs). A two-dimensional (valence and arousal) conceptualization of emotional mechanisms derived from the circumplex model of affects (CMAs) was adopted in this study. A distinguished switching mechanism was correlated between regular and chaotic dynamics when switching from neutral to arousal elicitation states [43]. Valenza et al. [106] reported the use of Lyapunov exponents to understand the instantaneous complex dynamics of the heart from the RR interval signals. The proposed method employed a high-order point-process nonlinear model for the analysis. The Volterra kernels (linear, quadratic, and cubic) were expanded using the orthonormal Laguerre basis functions. The instantaneous dominant Lyapunov exponents (IDLE) were estimated and tracked for the RRI time series. The results suggested that the proposed method was able to track the nonlinear dynamics of the autonomic nervous system- (ANS-) based control of the heart. Du et al. [107] reported the development of a novel Lyapunov exponent-based diagnostic method for the classification of premature ventricular contraction from other types of ECG beats.

HRV has been reported to be sensitive to both physiological and psychological disorders [108]. In recent years, HRV has been used as a tool in the diagnosis of the cardiac diseases. HRV is estimated by analyzing the RR intervals extracted from the ECG signals. The HRV analysis requires a sensitive tool, as the nature of the RR interval signal is chaotic and stochastic, and it remains very much controversial [108]. Researchers have proposed Lyapunov exponents as a means for improving the sensitivity of the HRV analysis. In earlier studies, Wolf et al. and Tayel and AlSaba had developed two algorithms for the estimation of the Lyapunov exponents [46, 108]. However, those methods were found to diverge while determining the HRV sensitivity. Recently, Tayel and AlSaba [108] proposed an algorithm known as Mazhar-Eslam algorithm that increases the sensitivity of the HRV analysis with improved accuracy. The accuracy was increased up to 14.34% as compared to Wolf's method. Ye and Huang [109] reported the estimation of Lyapunov exponents of the ECG signals for the development of an image encryption algorithm, which can provide security to images from all sorts of differential attacks. In the same year, Silva et al. [110] proposed the largest Lyapunov exponent-based analysis of the RR interval time series extracted from ECG signals for predicting the outcomes of HUTT.

### 4.3. Applications of Correlation Dimension in ECG Signal Analysis

The correlation dimension provides a measure of the amount of correlation contained in a signal. It has been used by a number of researchers for analyzing the ECG and the derived RRI time series in order to detect various pathological conditions [111, 112]. Bolea et al. proposed a methodological framework for the robust computation of correlation dimension of the RRI time series [113]. Chen et al. [114] used correlation dimension and Lyapunov exponents for the extraction of the features from the ECG signals for developing ECG-based biometric applications. The extracted ECG features could be classified with an accuracy of 97% using multilayer perceptron (MLP) neural networks [114]. Rawal et al. [115] proposed the analysis of the HRV during menstrual cycle using an adaptive correlation dimension method. In the conventional correlation dimension method, the time delay is calculated using the autocorrelation function, which does not provide the optimum time delay value. In the proposed method, the authors calculated the time delay using the information content of the RR interval signal. The proposed adaptive correlation dimension method was able to detect the HRV variations in 74 young women during the different stages of the menstrual cycle in the lying and the standing positions with a better accuracy than the conventional correlation dimension and the detrended fluctuation analysis methods. Lerma et al. [50] investigated the relationship between the abnormal ECG and the less complex HRV using correlation dimension. ECG signals (24 h Holter ECG signals as well as standard ECG signals) were acquired from 100 volunteers (university workers), among which 10 recordings were excluded due to the detection of >5% of false RR intervals. Examination of the rest 90 standard ECG signals by two cardiologists suggested 29 standard ECG signals to be abnormal. Estimation of the correlation dimensions suggested that the abnormal ECG signals were associated with reduced HRV complexity. Moeynoi and Kitjaidure analyzed the dimensional reduction of sleep apnea features by using the canonical correlation analysis (CCA). The sleep apnea features were extracted from the single-lead ECG signals. The linear and nonlinear techniques to estimate the variance of heart rhythm and HRV from electrocardiography signal were applied to extract the corresponding features. This study reported a noninvasive way to evaluate sleep apnea and used CCA method to establish a relationship among the pair data sets. The classification of the extracted features derived from apnea annotation was comparatively better than the classical techniques [116].

### 4.4. Applications of DFA in ECG Signal Analysis

It is a well-reported fact that the exposure to the environmental noise can result in annoyance, anxiety, depression, and various psychiatric diseases [117, 118]. However, noise exposure has also been reported to cause cardiovascular problems [118]. Chen et al. [114] proposed the DFA of the RR intervals during exposure to low-frequency noise for 5 min to detect the changes in the cardiovascular activity [119]. From the results, it could be summarized that an exposure to the low-frequency noise might alter the temporal correlation of HRV, though there was no significant change in the mean blood pressure and the mean RR interval variability. Kamath et al. reported the implementation of DFA for the classification of congestive heart failure (CHF) disease [120]. Short-term ECG signals of 20 sec duration, from normal persons and CHF patients, were subjected analysis using DFA. The receiver operating characteristics (ROC) curve suggested the suitability of the proposed method with an average efficiency of 98.2%. Ghasemi et al. reported the DFA of RR interval time series to predict the mortality of the patients in intensive care units (ICUs) suffering from sepsis [121]. In the proposed study, DFA was performed on the RR interval time series of the last 25 h duration of the survived and nonsurvived patients, who were admitted to the ICUs. The results suggested that the scaling exponent (*α*) was significantly different for the survived and the nonsurvived patients from 9 h before the demise and can be used to predict the mortality. Chiang et al. tested the hypothesis that cardiac autonomic dysfunction estimated by DFA can also be a potential prognostic factor in patients affected by end-stage renal disease and undertaking peritoneal dialysis. Total mortality and increased cardiac varied significantly with a decrease in the corresponding prognostic predictor DFA*α*1. DFA*α*1 (≥95%) was related to lower cardiac mortality (hazard ratio (HR) 0.062, 95% CI = 0.007–0.571, *P* = 0.014) and total mortality [122].

### 4.5. Applications of RQA in ECG Signal Analysis

RQA has found many applications in ECG signal analysis [123–125]. Chen et al. investigated the effect of the exposure to low-frequency noise of different intensities (for 5 min) on the cardiovascular activities using recurrence plot analysis [126]. The RR intervals were extracted from the ECG signals acquired during the noise exposure of intensities 70 dBC, 80 dBC, and 90 dBC. The change in the cardiovascular activity was estimated using RQA of the RR intervals. Based on the results, the authors concluded that RQA-based parameters can be used as an effective tool for analyzing the effect of the low-frequency noise even with a short-term RR interval time series.

Acharya et al. reported the use of RQA and Kolmogorov complexity analysis of RRI time series for the automated prediction of sudden cardiac death (SCD) risk [127]. In this study, the authors designed a sudden cardiac death index (SCDI) using the RQA and the Kolmogorov complexity parameters for the prediction of SCD. The statistically important parameters were identified using *t*-test. These statistically important parameters were used as inputs for classification using K-NN, SVM, decision tree, and probabilistic neural network. The K-NN classifier was able to classify the normal and the SCD classes with 86.8% accuracy, 80% sensitivity, and 94.4% specificity. The probabilistic neural network also provided 86.8% accuracy, 85% sensitivity, and 88.8% specificity. Based on the results, the authors proposed that RQA and Kolmogorov complexity analysis can be performed for the efficient detection of SCD. Apart from these studies, the RQA of the ECG signals has been widely studied for the detection of different types of diseases. A few RQA-based studies performed in the last 5 years for the diagnosis of different clinical conditions have been summarized in Table 2.

### 4.6. Applications of Poincaré Plot in ECG Signal Analysis

Ventricular fibrillation has been reported to be the most severe type of cardiac arrhythmia [131]. It results from the cardiac impulses that have gone berserk within the ventricular muscle mass and is indicated by complex ECG patterns [131]. Electrical defibrillation is used as an effective technique to treat ventricular fibrillation. Gong et al. reported the application of Poincaré plot for the prediction of occurrence of successful defibrillation in the patients suffering from ventricular fibrillation [132]. The Euclidean distance of the successive points in Poincaré plot was used to calculate the stepping median increment of the defibrillation, which in turn, was used to estimate the possibility of successful defibrillation. The testing of the proposed method was analyzed using the ROC curve, and the results suggested that the performance was comparable to the established methods for successfully estimating defibrillation.

Polycystic ovary syndrome (PCOS) is a common endocrine disease found in 5–10% of the reproductive women [133]. PCOS has been reported to be associated with cardiovascular risks due to its connection with obesity [134]. Saranya et al. performed the Poincaré plot-based nonlinear dynamical analysis of the HRV signals acquired from the PCOS patients to predict the associated cardiovascular risk [135]. The authors found that the PCOS patients had reduced HRV and autonomic dysfunction (in terms of increased sympathetic activity and reduced vagal activity), which might herald cardiovascular risks. Based on the results, the authors suggested that the Poincaré plot analysis may be used independently to measure the extent of autonomic dysfunction in PCOS patients. Some Poincaré plot-based studies performed in the last 5 years for the diagnosis of different clinical conditions have been given in Table 3.

### 4.7. Applications of Multiple Nonlinear Dynamical System Analysis Methods in ECG Signal Analysis

In the last few years, some researchers have also implemented multiple nonlinear methods simultaneously for the analysis of the ECG signals [42]. In some cases, the nonlinear methods have been used in combination with the linear methods [139]. Acharya et al. performed analysis of ECG signals using time domain, frequency domain, and nonlinear (i.e., Poincaré plot, RQA, DFA, Shannon entropy, ApEn, SaEn, higher-order spectrum (HOS) methods, empirical mode decomposition (EMD), cumulants, and correlation dimension) techniques for the diagnosis of coronary artery disease [140]. Goshvarpour et al. studied the effect of the pictorial stimulus on the emotional autonomic response by analyzing the nonlinear methods, that is, DFA, ApEn, and Lyapunov exponent-based parameters along with statistical measures of ECG, pulse rate, and galvanic skin response signals [141]. Karegar et al. extracted the nonlinear ECG features using the methods, namely, rescaled range analysis, Higuchi's fractal dimension, DFA, generalized Hurst exponent (GHE), and RQA for ECG-based biometric authentication [142]. The combination of different nonlinear methods for obtaining better performance was observed in the previously reported literature, but the studies prescribing superiority of one method in comparison to the other methods could not be found.

## 5. Conclusion

Most of the biosignals are nonstationary in nature, which often makes their analysis cumbersome using the conventional linear methods of signal analysis. This led to the development of nonlinear methods, which can perform a robust analysis of the biosignals [9]. Among the biosignals, the analysis of the ECG signals using nonlinear methods has been highly explored. The nonlinear analysis of the ECG signals has been investigated by many researchers for early diagnosis of diseases, human identification, and understanding the effect of different stimuli on the heart and the ANS. The current review dealt with the relevant theory, potential, and recent applications of the nonlinear ECG signal analysis methods. Although the nonlinear methods of ECG signal analysis have shown promising results, it is envisaged that the existing methods may be extended and new methods can be proposed to improve the performance and handle large and complex datasets.

## Figures and Tables

**Table 1:** Recent studies performed for arrhythmia detection using phase space analysis of ECG.

Types of arrhythmia	Classification method	Performance	Ref.
Atrial fibrillation, ventricular tachycardia, and ventricular fibrillation	Distribution of the attractor in the reconstructed phase space	85.7–100% sensitivity, 86.7–100% specificity, and 95.55% overall efficiency	[12]
Ventricular tachycardia, ventricular fibrillation, and ventricular tachycardia followed by ventricular fibrillation	Box-counting in phase space diagrams	96.88% sensitivity, 100% specificity, and 98.44% accuracy	[89]
Ventricular fibrillation and normal sinus rhythm	Neural network with weighted fuzzy membership functions	79.12% sensitivity, 89.58% specificity, and 87.51% accuracy	[90]
Atrial premature contraction, premature ventricular contraction, normal sinus rhythm, left bundle branch block, and right bundle branch block	K-nearest neighbour	99.42% sensitivity, 98.19% specificity, and 98.7% accuracy	[88]
Soon-terminating atrial fibrillation and immediately terminating atrial fibrillation	A genetic algorithm in combination with SVM	100% sensitivity, 100% specificity, and 100% accuracy	[91]

**Table 2:** Recent studies performed for the diagnosis of clinical conditions using RQA-based ECG analysis.

Clinical conditions	Classification method	Performance	Ref.
Atrial fibrillation, atrial flutter, ventricular fibrillation, and normal sinus rhythm	Decision tree, random forest, and rotation forest	98.37%, 96.29%, and 94.14% accuracy for rotation forest, random forest, and decision tree, respectively	[123]
Effect of the exposure to low-frequency noise of different intensities on the cardiovascular activities	Statistical analysis of RQA-based measures	Statistically significant parameters obtained with *p* value ≤ 0.05	[126]
Obstructive sleep apnea	A soft decision fusion rule combining SVM and neural network	86.37% sensitivity, 83.47% specificity, and 85.26% accuracy	[128]
Arrhythmia	Joint probability density classifier	94.83 ± 0.37% accuracy	[129]
Sudden cardiac death	K-NN, SVM, decision tree, and probabilistic neural network	86.8% accuracy, 80% sensitivity, and 94.4% specificity with K-NN classifier and 86.8% accuracy, 85% sensitivity, and 88.8% specificity with PNN	[127]
Atrial fibrillation	Unthresholded recurrence plots	72% accuracy	[130]

**Table 3:** Recent studies performed for the diagnosis of clinical conditions using Poincaré plot analysis.

Clinical conditions	Classification method	Performance	Ref.
Dilated cardiomyopathy	Multivariate discriminant analysis	92.9% sensitivity, 85.7% specificity, and 92.1% AUC	[136]
Preeclampsia	Multivariate discriminant analysis	91.2% accuracy	[137]
Polycystic ovary syndrome	Statistical analysis of Poincaré plot-based measures	Statistically significant parameters obtained with *p* value ≤ 0.05	[135]
Atrial fibrillation	SVM optimized with particle swarm optimization	92.9% accuracy	[138]
